# Expression Patterns of *TGF-β1*, *TβR-I*, *TβR-II,* and *Smad2* Reveal Insights into Heterosis for Growth of Hybrid Offspring between *Acanthopagrus schlegelii* and *Pagrus major*

**DOI:** 10.3390/genes15070945

**Published:** 2024-07-19

**Authors:** Xinran Du, Yue Zhao, Jingbo Li, Wenli Xie, Linna Lyu, Shuyin Chen, Chaofeng Jia, Jie Yan, Peng Li

**Affiliations:** 1 Herpetological Research Center, College of Life Sciences, Nanjing Normal University, Nanjing 210023, China; dessira@163.com (X.D.); yuezhao0222@163.com (Y.Z.); jingbo207@163.com (J.L.); xiewenli330@163.com (W.X.); llnlln1207@163.com (L.L.); yanjie@njnu.edu.cn (J.Y.); 2 Jiangsu Institute of Oceanology & Marine Fisheries, Nantong 226007, China; shuyinchen89@163.com (S.C.); chfjia@126.com (C.J.)

**Keywords:** *Acanthopagrus schlegelii*, *Pagrus major*, TGF-β1/Smads signal pathway, heterosis, mRNA expression, protein expression

## Abstract

TGF-β1/Smads is a classic signaling pathway, which plays important roles in the development process of organisms. Black porgy *Acanthopagrus schlegelii* and red porgy *Pagrus major* are valuable economic fishes, and their hybrid offspring show excellent heterosis traits. Yet the molecular regulation mechanism of the heterosis traits is less clear. Here, we explored the TGF-β1/Smads pathway’s molecular genetic information for heterosis in *A. schlegelii* ♂ × *P. major* ♀ (AP) and *A. schlegelii* ♀ × *P. major* ♂ (PA) in terms of growth and development. The mRNA expression levels of *TGF-β1*, *TβR-I*, *TβR-II*, and *Smad2* genes in different developmental stages of *A. schlegelii* were detected. Furthermore, the expression levels of *TGF-β1*, *TβR-I*, *TβR-II*, and *Smad2* genes in different tissues of adult (mRNA level) and larva (mRNA and protein level) of *A. schlegelii*, *P. major*, and their hybrids were determined by both real-time quantitative PCR and Western blot techniques. The results indicated the ubiquitous expression of these genes in all developmental stages of *A. schlegelii* and in all tested tissues of *A. schlegelii*, *P. major,* and its hybrids. Among them, the mRNA of *TGF-β1*, *TβR-I,* and *TβR-II* genes is highly expressed in the liver, gill, kidney, and muscle of black porgy, red porgy, and their hybrid offspring. There are significant changes in gene and protein expression levels in hybrid offspring, which indirectly reflect hybrid advantage. In addition, there was no correlation between protein and mRNA expression levels of Smad2 protein. The results provide novel data for the differential expression of growth and development genes between the reciprocal hybridization generation of black porgy and red porgy and its parents, which is conducive to further explaining the molecular regulation mechanism of heterosis in the growth and development of hybrid porgy.

## 1. Introduction

Black porgy (*Acanthopagrus schlegelii*) and red porgy (*Pagrus major*) are precious economic value fishes of the family Sparidae. They are all important artificial feeding fish of candidate species in different sea areas of China. The black porgy *A. schlegelii* has distinct dominant characteristics with delicious and nutritious flesh, and outstanding resistance to stress and a variety of diseases. *P. major* grows fast and has a beautiful body color. Hybridization is an important method for creating new varieties, which can combine the genomes of different fish species and play an important role in the evolution of genetic breeding organisms. Numerous studies on the hybridization of different fish species, e.g., *Sparus aurata* ♀ × *P. major* ♂ [1], *P. major* ♀ × *A. schlegelii* ♂ [2], and *P. major* ♀ × *S. sarba* ♂ [2], have accumulated rich experience in the crossbreeding of sea bream fish. In order to select excellent new varieties of marine aquaculture, there have been many studies on the crossbreeding of *A. schlegelii* × *P. major* [3]. There have been some studies on the karyotype, genetic similarity, stress resistance, or nutrients comparison of fish species/variety. After years of genetic selection and breeding of sea bream, Jiangsu Marine Fisheries Research Institute of China had obtained a new variety of sea bream fish with fast-growing, fertile, and strong resistance to stress [4]. The new variety (backcross) has a growth rate of more than 20% higher than that of the selfed offspring of *A. schlegelii* and *P. major*, and it has an obvious advantage in stress resistance [3]. Some studies have explored the growth and breeding conditions of the hybrid progeny of *A. schlegelii* × *P. major*, and they showed some economically valuable traits [5,6]. There is a prospect of breeding *A. schlegelii* ♂ × *P. major* ♀ (AP) and *A. schlegelii* ♀ × *P. major* ♂ (PA), but so far little is known about the molecular regulation mechanism of its excellent heterosis traits.

TGF-β (transforming growth factor β) is a superfamily of proteins with multiple functions, and plays a key role in cellular immunity, tissue loss, sex determination, and cell proliferation and differentiation [7]. TGF-β1 (transforming growth factor β 1) is a transforming factor first found in mesenchymal cells, which can inhibit the proliferation of epithelial cells [8]. Subsequent multitudinous members similar to the TGF-β1 structure were discovered, and collectively referred to as the TGF-β family [9]. TGF-β family members play a role by binding to specific cell surface receptors to trigger their biological effects, and their receptors can be divided into TβR-I~IV, of which TβR-I~III are widely expressed [7,10,11]. It has been found that the signal transduction of TGF-β1 is mostly mediated by a heterotetramer composed of TβR-I (transforming growth factor β receptor 1) and TβR-II (transforming growth factor β receptor 2). TβR-I type (53~65 kDa) and TβR-II type (70~100 kDa) are glycoprotein signal transmission receptors [10]. Their cytoplasmic segment has serine/threonine kinase activity, and the affinity of interaction with TGF-β1 is 10 times compared to TGF-β3 [10]. In addition to the ligands and receptors of the TGF-β superfamily, the Smad protein is another key factor to TGF-β1/Smads signaling pathway [12]. They are at the center of the entire signaling pathway and serve as a link. Specifically, when TGF-β1 is activated, it binds to the TβR-II receptor on the cell membrane, and then adsorbs the TβR-I and forms a heterologous complex with it on the cell membrane. Then, TβR-I is phosphorylated by TβR-II kinase. Activated TβR-I temporarily binds to R-Smads (Smad2 and Smad3 mainly) and phosphorylates the C-terminal Ser residue of R-Smads [13,14]. The activated R-Smads change their conformation and dissociate from the receptor, and then combine with Co-Smad (Smad4) to form a complex and transfer to the nucleus [15,16]. The activated complex is then combined with two types of DNA binding cofactors, namely co-repressor or co-activator, to determine the transcriptional activity of target genes, thereby regulating various cellular responses [17].

Previous research on TGF-β and its receptors in mammals revealed the significant importance of the TGF-β system in cell differentiation and individual growth [18,19,20]. In recent years, various studies have focused on the TGF-β system in vertebrates. Fish have their unique living environment and special significance in evolutionary history. It has been discovered that the TGF-β1/Smads signaling pathway exists in fish and has similar functions to that in mammals [21,22,23,24,25]. To analyze the regulatory effects of the growth-related genes and pathways of the hybrid offspring of marine black porgy and red porgy, and to further explore the molecular mechanism of sea bream heterosis, here we explore the expression patterns of key genes (*TGF-β1*, *TβR-I*, *TβR-II*, and *Smad2*) in TGF-β1/Smads pathway of *A. schlegelii*, *P. major*, *A. schlegelii* ♂ × *P. major* ♀ (AP), and *P. major* ♂ × *A. schlegelii* ♀ (PA) in terms of growth and development.

## 2. Material and Methods

### 2.1. Ethics Statement

Animal welfare and experimental procedures were approved by the Institutional Animal Care and Use Committee of Nanjing Normal University [SOXR (Jiangsu) 2020–0047 and IACUC-20220258]. The author confirms that at any experimental stage of this study, animals did not suffer unnecessary pain.

### 2.2. Fish Sampling

The samples were taken randomly from healthy adult individuals of one-, two-, and three-year-old *A. schlegelii*, one-year old *P. major*, and their hybrid offspring (*A. schlegelii* ♂ × *P. major* ♀ (AP) and *A. schlegelii* ♀ × *P. major* ♂ (PA)) that were farmed in the Lvshi Base of Jiangsu Provincial Institute of Marine Fisheries, Nantong, China. Three fish of each kind were used (18 in total). Eight tissue samples (5~30 mg for each sample) of brain, muscle, heart, gill, liver, kidney, intestine, and gonad from each kind were put into 1.5 mL tubes with RNA*later* RNA Stabilization Reagent (QIAGEN, Hilden, Germany, No. 76104), respectively. Each tissue sample was divided into 3 groups for repeated experiments.

An additional eight surviving larvae (30 day) (32 in total) of *A. schlegelii*, *P. major*, AP, and PA were collected and put into liquid nitrogen for 2 min, respectively, then frozen at −80 °C in a sealed centrifuge tube for total RNA (to further compare the expression level of genes) and protein extraction (to prepare for subsequent Western blot experiments).

All of the samples mentioned above were cultured under water temperature conditions of 20–25 °C and fed with benthic crustaceans and mollusks.

### 2.3. Sequence Analyses

This study used the program of the National Center for Biotechnology Information (NCBI) that uses BLAST (http://www.ncbi.nlm.nih.gov/blast/) (accessed on 20 January 2020) to analyze cDNA sequences and derived amino acid sequences and the ExPASy Molecular Biology server (http://us.expasy.org) (accessed on 3 March 2020). Predicting possible signal peptides and cleavage sites using SignalP 5.0 (http://www.cbs.dtu.dk/services/SignalP) (accessed on 17 March 2020). The transmembrane domain was predicted using TMHMM Server v. 2.0 (http://www.cbs.dtu.dk/services/TMHMM-2.0) (accessed on 23 May 2020). Generate multiple alignments of amino acid sequences using the ClustalW program version 2.0.

### 2.4. RNA Extraction and Real-Time Polymerase Chain Reaction Detection

According to the product manual, TransZol™ Up Plus RNA Kit (TransGen, Beijing, China, No. ER501.) extracts total RNA from various tissues. A NanoDrop ND2000C spectrophotometer (NanoDrop Technologies, Wilmington, DE, USA) was used to determine the concentration and purity of total RNA. For all RNA samples, A260/A280 ratios were in the range 2.0–2.1, respectively. According to the manufacturer’s plan, PrimeScript™ RT Master Mix (TaKaRa, Dalian, China, No. RR036A) was used to perform reverse transcription polymerase chain reaction to synthesize complementary DNA (cDNA). With *β-actin* as the internal control, fluorescence real-time quantitative polymerase chain reaction (qPCR) was used to detect the relative mRNA expression levels of *TGF-β1*, *TβR-I*, *TβR-II*, and *Smad2* in various tissues. All primers for the qPCR amplification of each gene are listed in Table 1. The PCR conditions were as follows: 94 °C for 5 min, 34 cycles of 94 °C for 30 s, 52 °C for 30 s, and 72 °C for 1 min.

### 2.5. Western Blot

Proteins from larvae were prepared using radioimmunoprecipitation assay buffer. Protein samples were subjected to concentration determination and immunoblot assay with TGF-β1 antibody (sabbiotech, College Park, MA, USA, No. 29701), TβR-I polyclonal antibody (sabbiotech, USA, No. 55035), anti-Smad2 antibody (abcam, Cambridge, UK, No. ab40855), and anti-TGF β Receptor II antibody (abcam, UK, No. ab184948). Anti-β Actin antibody (abcam, UK, No. ab8227) was used as the internal control.

### 2.6. Statistical Analyses

All data are represented as the mean ± SEM of the biological replicates shown. All data were verified for the data’s normality and homoscedasticity using R 4.4.3 software for the verification of “shapiro. test” and “bartlett. test”. Statistical analysis was performed using SPSS (version 21.0) statistical software (Chicago, IL, USA, SPSS Inc.). Student’s *t* tests were used for the comparison between two groups. For multiple group designs, after confirming the normal distribution of data, a two-way analysis of variance (two-way ANOVA) is used, and the results of ta wo-way analysis of variance (two-way ANOVA) were evaluated using the “kruskal. test” in R 4.4.3 software using nonparametric tests, followed by LSD post hoc testing. *α* < 0.05 was considered to be statistically significant.

## 3. Results

### 3.1. Sequence Analysis of AsTGF-β1, AsTβR-II, and AsSmad2

As shown in Supplementary Figure S1, the predicted protein of AsTGF-β1 (TGF-β1 from *A. schlegelii*) contains 379 amino acids, including a signal peptide of 19 amino acids and RXXR cleavage sites recognized by the protease KEX-furin, which cleaves peptides to produce prepeptides (LAPs) and mature peptides of 112 amino acids. The furin cleavage site is located anterior to the mature peptide of AsTGF-β1. Identify a characteristic RGD integrin-binding site in the precursor region. The mature peptide also contains nine conserved cysteine residues. The predicted AsTβR-II (TβR-II from *A. schlegelii*) contains 577 amino acids. A signal peptide of 26 amino acids was predicted at the N-terminus, and a transmembrane domain. In the extracellular domain, 11 conserved cysteines were found. The intracellular domain contained a predicted serine/threonine kinase domain. Characteristically, conservative KD domains also exist in the AsTβR-II (Supplementary Figure S2). As shown in Supplementary Figure S3, the predicted protein of AsSmad2 (Smad2 from *A. schlegelii*) contains 470 amino acids. A comparison between AsSmad2 and Smad2 in other species reveals that the DWA domain and DWB domain are conserved.

### 3.2. Expression of AsTGF-β1, AsTβR-I, AsTβR-II, and AsSmad2 mRNA in Different Developmental Stages of A. schlegelii

**Figure 1:**
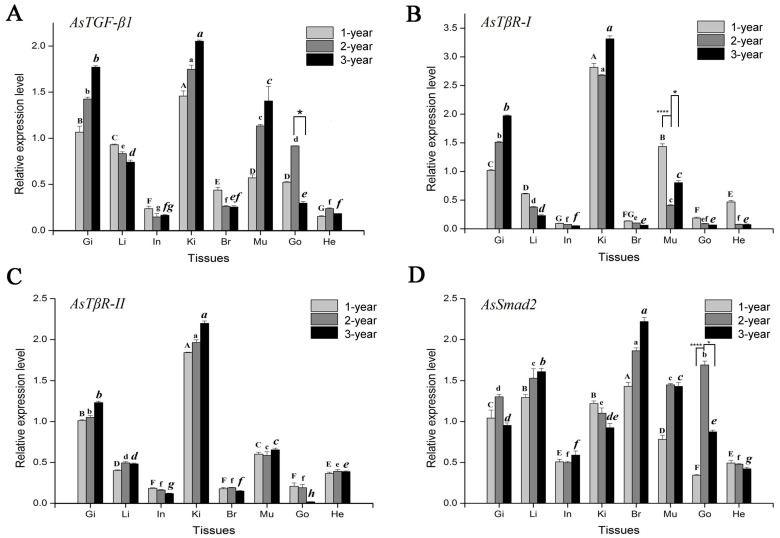
Relative expression of *AsTGF-β1* (**A**), *AsTβR-I* (**B**), *AsTβR-II* (**C**), and *AsSmad2* (**D**) mRNA in different developmental stages of *A. schlegelii.* Tissues are removed from the healthy *A. schlegelii,* and the abbreviations are as follows: Gi, gill; Li, liver; In, intestine; Ki, kidney; Br, brain; Mu, muscle; Go, gonad; and He, heart. Bars represent mean ± SEM (*n* = 3). Different letters indicate statistically significant differences. Same letter format represents statistically significant differences between the same groups. (*p* < 0.05); “*” indicates statistically significant differences (*p* < 0.05); and “****” indicates statistically significant differences (*p* < 0.0001).

The results from qPCR detection show that the *AsTGF-β1* gene of *A. schlegelii* is expressed in all tested developmental stages (Figure 1A). Among them, in the gill, kidney, and muscle tissues of *A. schlegelii*, the mRNA expression of the *AsTGF-β1* gene is relatively high and has a positive correlation with age. In the gonad, the expression level of *AsTGF-β1* mRNA in the 2-year-old *A. schlegelii* was higher than that in the 1-year-old and 3-year-old *A. schlegelii*, and the gonads of the 2-year-old *A. schlegelii* show significant differences compared to the 3-year-old *A. schlegelii* (as shown by “*” in Figure 1A). In the intestine, brain, and heart tissues of *A. schlegelii*, the *AsTGF-β1* gene has a relatively low mRNA expression level.

The mRNA expression patterns of *AsTβR-I* (*TβR-I* from *A. schlegelii*) and *AsTβR-II* at different developmental stages were detected; the results show that *AsTβR-I* and *AsTβR-II* mRNA are expressed at different developmental stages (Figure 1B,C). The mRNA expression of *AsTβR-I* and *AsTβR-II* in the gill and kidney has an upward trend with the age of *A. schlegelii*. The mRNA expression of *AsTβR-I* did not change significantly in the liver, intestine, and brain at different periods/ages. The mRNA expression of the AsTβR-I gene in the muscles of the 2-year-old *A. schlegelii* was significantly lower than its level in the 1-year-old and 3-year-old *A. schlegelii* (*p* < 0.05) (as shown by “*” in Figure 1B).

As shown in Figure 1D, the results of qPCR detection showed that the *AsSmad2* gene is expressed in the gill, liver, intestine, kidney, brain, muscle, gonad, and heart in *A. schlegelii*. In the liver and brain tissue, the mRNA expression level of *AsSmad2* increases with the age of *A. schlegelii*. Moreover, the mRNA expression level of the *AsSmad2* gene in the gonads in the 2-year-old *A. schlegelii* was significantly higher than that in the 1-year-old and 3-year-old *A. schlegelii* (*p* < 0.05) (as shown by “*” in Figure 1D). In the intestine and heart, the mRNA expression of *AsSmad2* did not change significantly among 1-year-old, 2-year-old, and 3-year-old *A. schlegelii*.

### 3.3. Expression of TGF-β1, TβR-I, TβR-II, and Smad2 mRNA in Different Tissues of A. schlegelii, P. major, and Their Hybrids

**Figure 2:**
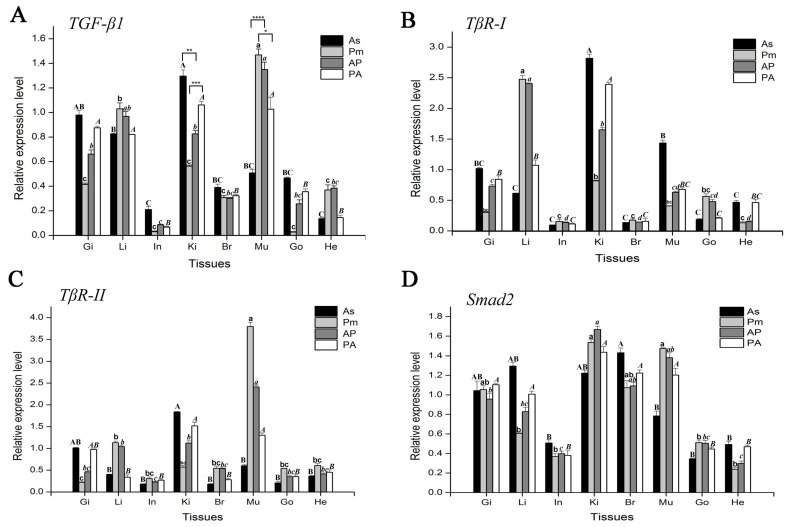
Expression of TGF-β1 (**A**), TβR-I (**B**), TβR-II (**C**), and Smad2 (**D**) mRNA in different tissues of *A. schlegelii*, *P. major*, and their hybrids. Tissues are removed from the healthy *A. schlegelii,* and the abbreviations are as follows: Gi, gill; Li, liver; In, intestine; Ki, kidney; Br, brain; Mu, muscle; Go, gonad; and He, heart. Bars represent mean ± SEM (*n* = 3). Different letters indicate statistically significant differences. Same letter format represents statistically significant differences between the same groups. (*p* < 0.05); “*” indicates statistically significant differences (*p* < 0.05); “**” indicates statistically significant differences (*p* < 0.01); “***” indicates statistically significant differences (*p* < 0.001); and “****” indicates statistically significant differences (*p* < 0.0001).

The results of qPCR detection showed that *TGF-β1* genes of *A. schlegelii*, *P. major,* and their hybrid progeny were expressed in all tested tissues. In the gill, liver, kidney, and muscle tissues of *A. schlegelii*, *P. major*, AP, and PA, the *TGF-β1* gene has a higher mRNA expression level, while a relatively low level of mRNA expression was detected in the intestines, brain, gonads, and heart. Moreover, the mRNA expression levels of the *TGF-β1* gene in the AP and PA were mostly between the two parents. And in the liver, kidney, muscle, and heart, the expression pattern of *TGF-β1* mRNA in the offspring is closer to that of the female parent. The *TGF-β1* mRNA expression level of AP is closer to that of *P. major*, while in the PA it is closer to that of *A. schlegelii*. In particular, there is a significant difference, in the kidney and muscle, in the expression pattern of *TGF-β1* mRNA between hybrid offspring and the male parent (as shown by “*” in Figure 2A).

Furthermore, *TβR-I* and *TβR-II* genes were expressed in the gill, liver, intestine, kidney, brain, muscle, gonads, and heart of *A. schlegelii*, *P. major,* and their hybrids. Using the mRNA expression level in the gill tissue of *A. schlegelii* as a reference, the *TβR-I* gene has relatively higher mRNA expression levels in the liver, kidney, and muscle of *A. schlegelii*, *P. major,* and their hybrids. *TβR-I* shows relatively higher mRNA expression levels in the liver, kidney, and muscle of *A. schlegelii*, *P. major,* and their hybrids, and relatively lower mRNA expression levels in the intestine, brain, gonad, and heart. However, *TβR-II* had a relatively higher mRNA expression in the kidneys and muscles of *A. schlegelii*, *P. major,* and their hybrids, and lower mRNA expression in the liver. It can be seen that the expression levels of *TβR-I* and *TβR-II* genes are not completely consistent. In addition, the expression levels of *TβR-I* and *TβR-II* genes in the hybrid offspring of *A. schlegelii* and *P. major* are mainly between the two parents (Figure 2B,C).

*Smad2* were expressed omnipresently in each tissue tested in *A. schlegelii*, *P. major,* and their hybrid progeny. *Smad2* mRNA expression levels are relatively lower in the intestines and hearts of *A. schlegelii*, *P. major,* and their hybrids, and relatively higher expression levels in other tissues tested. In addition, the mRNA expression of the *Smad2* gene in AP and PA was mostly between the expression levels of the two parents (Figure 2D).

### 3.4. The Expression of TGF-β1, TβR-I, TβR-II, and Smad2 Genes and Proteins in Larvae of A. schlegelii, P. major, AP, and PA

**Figure 3:**
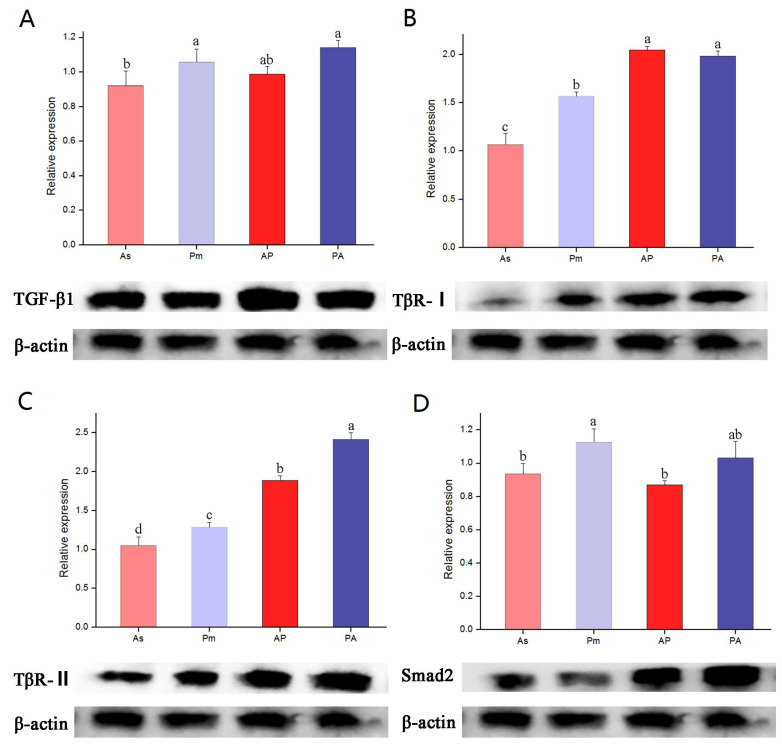
The gene expression and protein expression of TGF-β1 (**A**), TβR-I (**B**), TβR-II (**C**), and Smad2 (**D**) in larvae of *A. schlegelii*, *P. major*, AP, and PA. Different letters indicate statistically significant differences (*p* < 0.05).

The qPCR detection showed that the expression levels of *TGF-β1* mRNA in AP and PA were significantly higher than that of *A. schlegelii*, and the expression levels of *TβR-I* and *TβR-II* mRNA in the offspring were higher than that of the parent (*p* < 0.05). The mRNA expression of the *Smad2* gene in AP and PA tended to be different than that of their maternal parent. The Western blot detection showed that the protein expression of TGF-β1, TβR-I, TβR-II, and Smad2 in the hybrid offspring was higher than that of the parent, which was basically the same as the mRNA expression of *TGF-β1*, *TβR-I,* and *TβR-II*. The mRNA expression of the *Smad2* gene in the orthogonal progeny was significantly lower than that of *P. major* and PA, so there was no positive correlation between the expression levels of Smad2 protein and *Smad2* mRNA (Figure 3).

## 4. Discussion

TGF-β is a multifunctional cytokine that initiates many important processes within the adaptive and innate immune response [26]. Once bound by its receptor, the heteromeric receptor serine/threonine kinase complex recruits downstream effector protein Smad for signal transduction (for a diagram of TGF-β1/Smads pathways, see Supplementary Figure S5). Once phosphorylated by the receptor complex, the Smads mediate signals by oligomerization and migration to the nucleus, where they act as transcription factors affecting gene expression [15,27]. The hybrid offspring from *P. major* ♂ × *A. schlegelii* ♀ and *A. schlegelii* ♂ × *P. major* ♀ have been successfully farmed in an attempt to develop new marine aquaculture varieties with optimal target traits (for a photo of *A. schlegelii*, *P. major,* and their hybrid offspring, see Supplementary Figure S4). To analyze the molecular mechanism of different growth traits between AP and PA, we have identified and characterized four major elements of such a putative TGF-β signaling complex in sea bream TGF-β1, TβR-I, and TβR-II serine/threonine kinases and Smad2. We have also investigated their expression patterns in different tissues and developmental stages. From the perspective of aquaculture conditions for porgy, there are strict controls on abiotic factors such as temperature, water visibility, seawater salinity, pH value, and dissolved oxygen. In terms of biotic factors, black and red snappers are omnivorous fish that mainly feed on benthic crustaceans, mollusks, echinoderms, turtles, shrimp, and algae. In order to avoid the influence of environmental factors among individuals, this experiment standardized the expression levels of *A. schlegelii*, *P. major*, and their hybrid offspring. The aforementioned factors may alter the expression levels of *A. schlegelii*, *P. major*, and their hybrid offspring. In the future, we can better elaborate on expression levels in *A. schlegelii*, *P. major*, and their hybrid offspring from these perspectives.

The expression of *TGF-β1* mRNA in different developmental stages of *A. schlegelii* showed that it was significantly increased in the gonads in 2-year-old *A. schlegelii*. In addition, the *TGF-β1* mRNA expression level in the kidney of *A. schlegelii* increased year by year. In teleosts, such as grass carp and zebrafish, the kidney is the site where main B cell production and binding occurs [28,29]. In rainbow trout, the developing B cells mature in the head kidney and migrate to the activation site of the spleen or metanephric kidney, and the gill also acts as an immune organ in teleosts [30]. Therefore, the mRNA expression of the *AsTGF-β1* gene was highly expressed in the kidney and gill, suggesting that *AsTGF-β1* may have a potential function in the immune system of *A. schlegelii*, and the immune capacity of *A. schlegelii* increased significantly with age. Similar to *A. schlegelii*, *TGF-β1* mRNA expression level was highest in the kidney of *P. major* and its offspring, respectively. Our results were similar to those of salmon [31], rainbow trout [32], carp and goldfish [33,34], suggesting that TGF-β1 may play a major role in teleost immune regulation. *TGF-β1* mRNA expression in liver is higher in *A. schlegelii*, *P. major,* and their hybrid offspring. Hobbie reports that with dimethyl phosphite with ammonium nitrate medaka (*Oryzias latipes*), the expression of *TGF-β1* mRNA in liver was increased [35]. Moreover, with the increase in dimethyl phosphite ammonium nitrate concentration processing, TGF-β1 protein levels in the gallbladder epithelial cells, intermediate cells, immature liver cells, and a small amount of mature hepatocytes were increased, which shows TGF-β1 is associated with liver function. However, *TGF-β1* expression was not detected in the liver of rainbow trout and grass carp [32,34], and the reason for this difference needs further investigation. In the liver and heart, the hybrid inherited the maternal growth traits, and compared with the paternal heterosis these are more obvious. From the anatomical analyses of gill and gonad, it can be seen that the characteristics of the male parent are also obtained in the hybridization, and are significantly increased compared to those of *P. major*, highlighting the heterosis [3]. Therefore, it can be confirmed that AP and PA bred by *P. major* and *A. schlegelii* in this study combined the excellent characteristics of their parents and had heterosis.

The mRNA expression levels of the TGF-β receptor gene were detected in all sampling tissues. It has been suggested that TβR-I and TβR-II are necessary for TGF-β1 signaling. Therefore, two kinds of receptors are widely distributed and TβR-I and TβR-II may involve the fish TGF-β superfamily to mediate a large number of biological responses. A higher expression of *TβR-I* and *TβR-II* mRNA in kidney and gills prompt the meaning of these receptors in the immunology response. Both receptors were found to be highly expressed in muscle. Research showed that *TβR-I* and *TβR-II* mRNA are highly expressed in the skeletal muscle of red porgy *P. major* and its expression is increased after starvation in young fish [36]. It is speculated that the TGF-subtypes in fish all act through the same TGF-β receptors and are involved in regulating muscle differentiation and proliferation. In *A. schlegelii*, *TβR-I* and *TβR-II* mRNA in muscle increased with age, suggesting that they are involved in regulating muscle differentiation and proliferation, related to growth and development. Moreover, the 1-year-old and 2-year-old porgy includes the period with the fast growth rate in the whole growth stage. The 3-year-old porgy has achieved full adult and complete sexual maturity. As mentioned above, immune regulation and muscle proliferation and differentiation are age-related physiological differences that may alter the expression patterns of the TGF-β gene family. A study has shown that TβR-I and TβR-II are involved in the formation of different regions and different cells in the mammalian central nervous system (CNS) [37]. But in our study, the mRNA expression level of *TβR-I* and *TβR-II* in the brain tissue of black porgy and red porgy is not very high. Due to differences in species, *TβR-I* and *TβR-II* mRNA expression shows different patterns. Considering that TβR-II signaling requires TβR-I, and TβR-I and their ligand binding require TβR-II, it can be expected that the two receptor forms will have similar expression patterns. But *TβR-I* and *TβR-II* mRNA expression is not completely consistent in some tissues (e.g., heart), we speculate that receptors differently expressed play a role in physiological response in the TGF-β regulation. Previous research indicated that TβR-I and TβR-II distribution and the percentage change in the TβR-I differences play an important role in regulating cell proliferation [10], while TβR-I and TβR-II mediate different signaling pathways, which is the foundation of its different proliferation-regulating effect: TβR-I mainly adjusts the Smad signaling pathway system and has the role of cell proliferation inhibition. Nevertheless, TβR-II has adjusted the non-Smad signaling system and has the effect of promoting cell proliferation. In addition, the fact that *TβR-I* and *TβR-II* are expressed between the parents is a powerful evidence of heterosis.

Fluorescence quantitative PCR detection revealed that the *Smad2* mRNA expression level was higher in the muscle tissues of *A. schlegelii*, *P. major,* and their hybrid offspring, indicating its significance for the muscle growth and development of sea bream. Several proteins in the Smad family have been shown to be involved in the control of muscle growth in human (or mice) [38]. Transforming growth factor β (TGF-β) family ligands such as Myostatin, TGF-β combined into muscle cells activation ActRIIA, ActRIIB II type receptors, and TβR-II, further raise the tyrosine kinase activation type I receptor (ALK4, ALK5, and ALK7), causing Smad2 and Smad3 (Smad2/Smad3) phosphorylation. Smad2/Smad3 and Smad4 form transcriptional regulatory complexes that enter the nucleus and reduce the expression of a range of muscle growth-related genes by influencing chromatin structure [10]. On the other hand, Bone Morphogenetic Protein (BMP) binds to the cell membrane receptor BMPRII or ActRIIA/ActRIIB, raises and activates other activation I receptors (ALK2 ALK3, and ALK6), and causes the Smad family of Smad1/5/8 phosphorylation and activation [39]. The activated Smad1/5/8 complex and activated Smad2/3 have opposite effects on muscle growth. It binds to Smad4 in a similar manner to Smad2/3 to form the Smad1/5/8-Smad4 transcriptional regulatory complex, which improves the expression of genes related to cell growth and differentiation, including muscle growth-related genes [30]. A study using transgenic mouse models revealed that the balance between muscle atrophy and muscle overgrowth depended on whether Smad4 was recruited by the Myostatin signaling pathway or by the BMP signaling pathway [39]. In addition, the level of *TGF-β1*, *TβR-I*, *TβR-II,* and *Smad2* gene mRNA of AP expressed between the *A. schlegelii* and *P. major* was studied. Furthermore, the mRNA expression of the four genes in AP is closer to that of *P. major*, while the mRNA expression level of the four genes in PA is closer to that of *A. schlegelii*. These results suggest that the TGF-β1/Smads signal transduction pathway of key gene mRNA expression in the hybrid offspring may be passed down. Jiang et al. (2009) pointed out that the hybrid bream F1 generation obtained the characteristics of a faster growth rate of the female bream, which was significantly faster than that of the male bream, showing the heterosis [40]. Yan et al. (2010) compared the suffocating point difference between hybrid bream and parent bream, and concluded that the suffocating point of hybrid bream was closer to the mother and the maternal inheritance was obtained [41]. It was concluded from the temperature test of this study that the hybrid F obtained the excellent characteristics of the male *A. schlegelii* with wide adaptability to temperature, and the suitable temperature range was increased. From the comparative analyses of growth phenotypic traits, the hybrid F1 obtained the characteristics of the fast growth rate of female *P. major*, which was significantly faster than that of male *A. schlegelii*, highlighting the heterosis. So it can be concluded that hybrid F1 inherits and combines the excellent characteristics of *P. major* and *A. schlegelii*, and has the heterosis. There is research indicating a genetic heterozygosity enhancement and band spectrum increase in a RAPD analysis of hybrid F1 [42]. Thus, the hybridization of *P. major* and *A. schlegelii* can enrich the genetic structure, individual genes appear as new genetic types, and excellent varieties have been chosen and bred. Therefore, cross breeding can add new and better genetic traits to an existing variety, or combine two or more types with different good traits into a new and better type. Proper hybridization can not only simply combine the superior traits of different types of parents, but can also produce traits superior to those of the parents that have never been seen before [43,44]. Hybridization is an important way to create variation.

*TGF-β1*, *TβR-I*, *TβR-II,* and *Smad2* genes were expressed in the larvae of *A. schlegelii*, *P. major,* and their hybrid offspring, reflecting the universality of the TGF-β1/Smads signaling pathway in teleosts. The mRNA expression levels of *TGF-β1*, *TβR-I,* and *TβR-II* in the hybrid progeny all showed the hybridization superiority, and were generally consistent with the expression patterns of their respective corresponding proteins. The mRNA expression pattern of Smad2 in progeny was closer to that of the mother, that is, the expression level of AP Smad2 was consistent with that of *A. schlegelii*, and lower than that of *P. major* and PA. Moreover, the protein expression of AP Smad2 was lower than that of PA and higher than that of parents *A. schlegelii* and *P. major,* respectively. There was no positive correlation between Smad2 protein and mRNA expression level, which may be related to post-transcriptional regulation and protein degradation, suggesting that there are other regulatory ways to regulate signal transduction in the TGF-β1/Smads signaling pathway. In terms of early ontogenetic development, the expression pattern of the TGF-β gene family in hybrid offspring is closer to that of the maternal parent. Therefore, the feeding regime during the larval breeding period of hybrid offspring can be cultivated according to the maternal feeding mode, which is speculated to better improve the quality of porgy breeding. In recent years, it has been found that the ubiquitin-proteasome pathway (UPP) is an important pathway to regulate protein degradation and function [45], which proves that Smad protein may be regulated by SMURF-2 in this experiment. The Ubiquitin Proteasome Pathway (UPP) is an important pathway that regulates protein degradation and function. Under the catalysis of ubiquitin-proteinligase (E3), substrate protein ubiquitination, this ubiquitin-proteinligase process is repeated to form a polyubiquitin chain, which makes substrate protein more easily hydrolyzed by protease [46]. Smad ubiquitination regulatory factor 1 (Smurf-1) and Smad ubiquitination regulatory factor 2 (Smurf-2) are two kinds of E3 ligases that regulate the ubiquitination and degradation of Smad proteins [27]. Smurf-1 can independently induce the ubiquitination and degradation of Smad1 and Smad5. If there is a mutation, it will lead to the suppression of the BMP signal transduction pathway and affect the embryonic development [47]. Smurf-2, induced by TGF-β1, can form complexes with Smad2 and eventually degrade it. Smurf-2 can also degrade TGF-β1 receptor complexes of Smad1, Smad5, Smad7, and transcriptional co-repressors. A study has revealed that the abnormal expression of Smurf-1 and Smurf-2, which cannot inhibit TGF-β1 signal transduction, will promote the occurrence and development of scleroderma, a fibrous disease [48]. After 8 weeks of liver fibrosis in mice, the expression level of smurf-2 in the liver was decreased and the level of Smad2 was up-regulated, which also proved from the side that the Smurf-2 protein of hybrid bream in this experiment may be regulated by Smurf-2 [49]. It has been shown that the loss of the TGF-β1 signal can lead to abnormal cell proliferation and carcinogenesis. Many human tumors have been found to contain either inactivated mutations in TGF-β1 receptors (TβR-I and TβR-II), or mutations in Smad proteins that antagonize TGF-β1-induced growth inhibition [50]. In this study, Smad protein may be regulated by Smurf-2, but how this pathway affects the Smad2 protein needs further research.

## 5. Conclusions

This study explored the TGF-β1/Smads pathway’s molecular genetic information for heterosis in *A. schlegelii* ♂ × *P. major* ♀ (AP) and *P. major* ♂ × *A. schlegelii* ♀ (PA) in terms of growth and development. The ubiquitous expression was detected in all developmental stages and in all different tissues tested from *A. schlegelii*, *P. major,* and its hybrids. The main results are as follows: (1) Key genes in TGF-β1/Smads pathways (TGF-β1, TβR-I, TβR-II, and Smad2) are expressed in various developmental stages of black porgy and in all tissues of black porgy, red porgy, and their hybrid offspring. (2) The mRNA expression levels of key genes in TGF-β1/Smads pathways (TGF-β1, TβR-I, TβR-II, and Smad2) in hybrid offspring are mostly between those in parents *A. schlegelii* and *P. major*, respectively (Figure 2). (3) The mRNA expression of these two receptors, TβR-I and TβR-II, is not entirely consistent in certain tissues of porgy (e.g., heart), indicating that a differential expression of mRNA in TβR-I and TβR-II may be involved in regulating TGF-β physiological effects in response. (4) There is no positive correlation between the expression level of Smad2 protein and Smad2 mRNA, which indicates that Smad2 may have post transcriptional regulation.

The above results provide some basic data for the functional gene expression of trait differences between progeny and parents obtained by the intergeneric hybridization of Sparidae. Our study provides insights into the cross breeding and popularization of porgy breeding, and are theoretically conducive to further explaining the molecular control mechanism of heterosis in the growth and development of hybrid porgy.

## Figures and Tables

**Table 1:** Primers used for fluorescent real-time quantitation PCR detection.

Genes	Forward Primer (5′-3′)	Reverse Primer (3′-5′)
*AsTGF-β1*	TTGGCACCATGAAGCTGGTA	TGAGGCTCTGTTGGTAAGCG
*PmTGF-β1*	TGGCTGTCCTTCGATGTCAC	TGGCTGTCCTTCGATGTCAC
*APTGF-β1*	CTCATCAGGCTGGAGACGAG	ATGTCGGTCTGGACCTCAGT
*PATGF-β1*	ACTGAGGTCCAGACCGACAT	TCAGCAGGCGGTAATCTCCA
*AsTβR-I*	CTCAGGCTCTGGATCAGGTC	TCTCCTGGAGGATGATGGTC
*AsTβR-II*	CATAACCCCACTCTGCCACT	GCCACAGACCATCATGACAC
*PmTβR-I*	CACCATCGACATCGCTCCAA	CGTAGTAGGGCAGCTGGTAG
*PmTβR-II*	GACATGCTGCCTGTGTGACT	GCTGTACCCACCTGTCCACT
*APTβR-I*	CACCATCGACATCGCTCCAA	CGTAGTAGGGCAGCTGGTAG
*APTβR-II*	TGGTGCCTCTGCTGGTTATG	GCGTGGAGCTACTGTCTGAA
*PATβR-I*	CCTCAATGGACCACCCCTTC	TCCTCACCACGCCATTTACC
*PATβR-II*	TGATGTTCGACAAGGGAGCC	CGGTAGAAGTAGAAGGCGGC
*AsSmad2*	ACGCAAGCCCACGCATAC	TCTTCCAACCCAGGAGTCGTT
*Asβ-actin*	GGACCTGTATGCCAACACTG	TGATCTCCTTCTGCATCCTG
*PmSmad2*	ACCTTCACAGCCATCACGAG	GGTCTCCACCCTCTGGTAGT
*Pmβ-actin*	TATCGTCATGGACTCCGGTG	TGATGTCACGCACGATTTCC
*APSmad2*	GAGGCCAGCGATCAACAGAT	CTGGCTCCGAGTAAGTCACC
*APβ-actin*	CAGAGCGTGGCTACTCCTTC	CCATCTCCTGCTCGAAGTCC
*PASmad2*	CTCCAGCCGGTGACTTACTC	CTCCACAGTGGCATTCCTGT
*PAβ-actin*	GGCTACTCCTTCACCACCAC	CCGATGGTGATGACCTGTCC

## Data Availability

The original contributions presented in the study are included in the article/Supplementary Material, further inquiries can be directed to the corresponding author.

## Supplementary Materials

The following supporting information can be downloaded at: https://www.mdpi.com/article/10.3390/genes15070945/s1, Figure S1. Multiple alignment of the deduced amino acid (aa) sequences of TGF-β1. The multiple alignment was produced using ClustalW. The black box marked TGF-β1. RGD and RKRR sites of homologous amino acid sequences; “◆” marks proline and glycine, while “▼” represents cysteine residues. Figure S2. Multiple alignment of the deduced amino acid (aa) sequences of TβR-II. The multiple alignment was produced using ClustalW. Figure S3. Multiple alignment of the deduced amino acid (aa) sequences of Smad2. The multiple alignment was produced using ClustalW. Figure S4. Body shapes of *Acanthopagrus schlegelii*, *Pagrus major* and their hybrid offsprings. Figure S5. Diagram of TGF-β1/Smads pathways. References [51,52] are cited in the Supplementary Materials.

## References

[1] Gorshkov S., Gorshkova G., Hadani A., Gordin H., Knibb W. (2002). Chromosome set manipulations and hybridization experiments in gilthead seabream (*Sparus aurata*). II. Assessment of diploid and triploid hybrids between gilthead seabream and red seabream (*Pagrus major*). J. Appl. Ichthyol..

[2] Chen S., Zhang Z., Ji H., Li P., Zhao Y., Zhang Z. (2018). Cloning and expression analysis of Calmodulin from the hybrid F1 of *Acanthopagrus schlegelii* male × *Pagrus major* female and *P. major*. Mar. Fish..

[3] Chen S., Zhang Z., Liu H., Jia C., Xu J., Zhu F., Zhang Z., Ren Z. (2017). Backcross Breeding and Observation of Early Larva Development in Red Bream *Pagrosomus major* and Black Bream *Acanthopagrus schiegelii*. Fish. Sci..

[4] Yu Y.Y. (2012). Genetic Analysis of the Inter Genus Hybridization of *Pagrosomus major* (♀) and *Spraus macrocephalus* (♂). Master’s Thesis.

[5] Wang S.T. (2018). Effects of Salinity on the Antioxidant System of Black Porgy (*Acanthopagrus schlegelii*) and Comparison of Fatty Acid Composition between Hybrid Snapper and Its Parents. Master’s Thesis.

[6] Wang S.T., Li L., Chen S.Y., Zhang Z.Y., Yin S.W., Zhu F., Jia C.F., Liu H.L. (2016). Lipid Content and Fatty Acid Profile of *Acanthopagrus schlegeli*, *Pagrosomus major* and Their Hybrids. Food Sci..

[7] Chen J.L., Colgan T.D., Walton K.L., Gregorevic P., Harrison C.A. (2016). The TGF-β Signalling Network in Muscle Development, Adaptation and Disease. Adv. Exp. Med. Biol..

[8] Shi Y. (2001). Structural insights on Smad function in TGFβ signaling. BioEssays News Rev. Mol. Cell. Dev. Biol..

[9] Chen M., Lv Z., Huang L., Zhang W., Lin X., Shi J., Zhang W., Liang R., Jiang S. (2015). Triptolide inhibits TGF-β1-induced cell proliferation in rat airway smooth muscle cells by suppressing Smad signaling. Exp. Cell Res..

[10] Huang S.S., Huang J.S. (2005). TGF-β control of cell proliferation. J. Cell. Biochem..

[11] Massagué J., Blain S.W., Lo R.S. (2000). TGFβ signaling in growth control, cancer, and heritable disorders. Cell.

[12] Wrana J.L. (2002). Phosphoserine-dependent regulation of protein-protein interactions in the Smad pathway. Structure.

[13] Derynck R., Zhang Y., Feng X.H. (1998). Smads: Transcriptional activators of TGF-β responses. Cell.

[14] Mehra A., Attisano L., Wrana J.L. (2000). Characterization of Smad phosphorylation and Smad-receptor interaction. Methods Mol. Biol..

[15] Derynck R., Zhang Y.E. (2003). Smad-dependent and Smad-independent pathways in TGF-β family signalling. Nature.

[16] Shi Y., Wang Y.F., Jayaraman L., Yang H., Massagué J., Pavletich N.P. (1998). Crystal structure of a Smad MH1 domain bound to DNA: Insights on DNA binding in TGF-β signaling. Cell.

[17] Wrana J.L., Attisano L. (2000). The Smad pathway. Cytokine Growth Factor Rev..

[18] Lau M.T., Ge W. (2005). Cloning of Smad2, Smad3, Smad4, and Smad7 from the goldfish pituitary and evidence for their involvement in activin regulation of goldfish FSHβ promoter activity. Gen. Comp. Endocrinol..

[19] Frolik C.A., Dart L.L., Meyers C.A., Smith D.M., Sporn M.B. (1983). Purification and initial characterization of a type beta transforming growth factor from human placenta. Proc. Natl. Acad. Sci. USA.

[20] Müller F., Blader P., Rastegar S., Fischer N., Knöchel W., Strähle U. (1999). Characterization of zebrafish smad1, smad2 and smad5: The amino-terminus of smad1 and smad5 is required for specific function in the embryo. Mech. Dev..

[21] Johnson A.K., Harms C.A., Levine J.F., Law J.M. (2006). A quantitative real-time RT-PCR assay to measure TGF-β mRNA and its correlation with hematologic, plasma chemistry and organo-somatic indices responses in triamcinolone-treated Atlantic menhaden, *Brevoortia tyrannus*. Dev. Comp. Immunol..

[22] Kohli G., Hu S., Clelland E., Di Muccio T., Rothenstein J., Peng C. (2003). Cloning of transforming growth factor-β1 (TGF-β1) and its type II receptor from zebrafish ovary and role of TGF-β1 in oocyte maturation. Endocrinology.

[23] Tan Q., Zagrodny A., Bernaudo S., Peng C. (2009). Regulation of membrane progestin receptors in the zebrafish ovary by gonadotropin, activin, TGF-β and BMP-15. Mol. Cell. Endocrinol..

[24] Chen J.X., Yi X.J., Gu P.L., Gao S.X. (2019). The role of KDR in intrauterine adhesions may involve the TGF-β1/Smads signaling pathway. Braz. J. Med. Biol. Res..

[25] Dong J., Zhu Z., Cui G., Zhang Z., Yue J., Zhang Y., Yao X., Huo M., Wei J., Wang Q. (2021). Gastric cancer derived exosomes induce peritoneal mesothelial cell EMT through TGF-β1/Smads pathway to promote peritoneal metastasis. bioRxiv.

[26] Holopainen R., Tapiovaara H., Honkanen J. (2012). Expression analysis of immune response genes in fish epithelial cells following ranavirus infection. Fish Shellfish Immunol..

[27] Lee P.S.W., Chang C., Liu D., Derynck R. (2003). Sumoylation of Smad4, the common Smad mediator of transforming growth factor-β family signaling. J. Biol. Chem..

[28] Yu E., Xie J., Wang G., Yu D., Gong W., Li Z., Wang H., Xia Y., Wei N. (2014). Gene Expression Profiling of Grass Carp (*Ctenopharyngodon idellus*) and Crisp Grass Carp. Int. J. Genom..

[29] Zhan X.L., Ma T.Y., Wu J.Y., Yi L.Y., Wang J.Y., Gao X.K., Li W.S. (2015). Cloning and primary immunological study of TGF-β1 and its receptors TβR I/TβR II in tilapia (*Oreochromis niloticus*). Dev. Comp. Immunol..

[30] Rindom E., Vissing K. (2016). Mechanosensitive Molecular Networks Involved in Transducing Resistance Exercise-Signals into Muscle Protein Accretion. Front. Physiol..

[31] Lilleeng E., Penn M.H., Haugland O., Xu C., Bakke A.M., Krogdahl A., Landsverk T., Frøystad-Saugen M.K. (2009). Decreased expression of TGF-β, GILT and T-cell markers in the early stages of soybean enteropathy in Atlantic salmon (*Salmo salar* L.). Fish Shellfish Immunol..

[32] Maehr T., Wang T., González Vecino J.L., Wadsworth S., Secombes C.J. (2012). Cloning and expression analysis of the transforming growth factor-β receptors type 1 and 2 in the rainbow trout *Oncorhynchus mykiss*. Dev. Comp. Immunol..

[33] Haddad G., Hanington P.C., Wilson E.C., Grayfer L., Belosevic M. (2008). Molecular and functional characterization of goldfish (*Carassius auratus* L.) transforming growth factor beta. Dev. Comp. Immunol..

[34] Zhan Y., Jimmy K. (2000). Molecular isolation and characterisation of carp transforming growth factor β1 from activated leucocytes. Fish Shellfish Immunol..

[35] Hobbie K.R., DeAngelo A.B., George M.H., Law J.M. (2012). Neoplastic and nonneoplastic liver lesions induced by dimethylnitrosamine in Japanese medaka fish. Vet. Pathol..

[36] Cai Z., Gao C., Li L., Xing K. (2010). Bipolar properties of red seabream (*Pagrus major*) transforming growth factor-β in induction of the leucocytes migration. Fish Shellfish Immunol..

[37] Annes J.P., Munger J.S., Rifkin D.B. (2003). Making sense of latent TGFβ activation. J. Cell Sci..

[38] Heldin C.H., Miyazono K., ten Dijke P. (1997). TGF-β signalling from cell membrane to nucleus through SMAD proteins. Nature.

[39] Walsh D.W., Godson C., Brazil D.P., Martin F. (2010). Extracellular BMP-antagonist regulation in development and disease: Tied up in knots. Trends Cell Biol..

[40] Jiang H., Wu X. (2009). Comparative Study on Temperature Tolerance and Growth between *Pagrus major* (Temminck et Schlegel) and *Spraus macrocephalus* and F_1_ Hybrid. Mod. Fish. Inf..

[41] Yan F., Xu S., Gu J., Chen X., Lyu H., Jia C. (2010). Comparison of metabolic and excretion rates of young *Sparus macrocephalus* and young hybrid porgy. J. Oceanogr. Taiwan Strait.

[42] Lin M., Miao L., Li M.Y., Yu Y.Y., Xu W.T. (2014). Chromosome composition and AFLP analysis of *Pagrosomus major*, *Spraus macrocephalus* and the hybirds. J. Oceanogr. Chin. Version.

[43] Harms C.A., Howard K.E., Wolf J.C., Smith S.A., Kennedy-Stoskopf S. (2003). Transforming growth factor-β response to mycobacterial infection in striped bass *Morone saxatilis* and hybrid tilapia *Oreochromis* spp. Vet. Immunol. Immunopathol..

[44] Harms C.A., Kennedy-Stoskopf S., Horne W.A., Fuller F.J., Tompkins W.A. (2000). Cloning and sequencing hybrid striped bass (*Morone saxatilis* x *M. chrysops*) transforming growth factor-β (TGF-β), and development of a reverse transcription quantitative competitive polymerase chain reaction (RT-qcPCR) assay to measure TGF-β mRNA of teleost fish. Fish Shellfish Immunol..

[45] Zi Z., Chapnick D.A., Liu X. (2012). Dynamics of TGF-β/Smad signaling. FEBS Lett..

[46] Gaarenstroom T., Hill C.S. (2014). TGF-β signaling to chromatin: How Smads regulate transcription during self-renewal and differentiation. Semin. Cell Dev. Biol..

[47] Qin B.Y., Lam S.S., Correia J.J., Lin K. (2002). Smad3 allostery links TGF-β receptor kinase activation to transcriptional control. Genes Dev..

[48] Meng X.M., Nikolic-Paterson D.J., Lan H.Y. (2016). TGF-β: The master regulator of fibrosis. Nat. Rev. Nephrol..

[49] Attisano L., Wrana J.L. (2000). Smads as transcriptional co-modulators. Curr. Opin. Cell Biol..

[50] Xu J., Attisano L. (2000). Mutations in the tumor suppressors Smad2 and Smad4 inactivate transforming growth factor β signaling by targeting Smads to the ubiquitin-proteasome pathway. Proc. Natl. Acad. Sci. USA.

[51] Cao G. (2021). Comparative Research of Genetic Characterization in *Acanthopagrus schlegelii*, *Pagrus major* and Their Hybrid Offsprings. Master’s Thesis.

[52] Chen Y., Fan Y., Guo D.Y., Xu B., Shi X.Y., Li J.T., Duan L.F. (2020). Study on the relationship between hepatic fibrosis and epithelial-mesenchymal transition in intrahepatic cells. Biomed. Pharmacother..

