# Supplementary material for: Expression Patterns of TGF-β1, TβR-I, TβR-II, and Smad2 Reveal Insights into Heterosis for Growth of Hybrid Offspring between Acanthopagrus schlegelii and Pagrus major
Source: Genes (Basel). 2024 Jul 19;15(7):945. doi: 10.3390/genes15070945 (PMC11276220; doi:10.3390/genes15070945)
Supplement: Supplementary file 1 [file genes-15-00945-s001.zip › genes-3086156-supplementary.pdf]

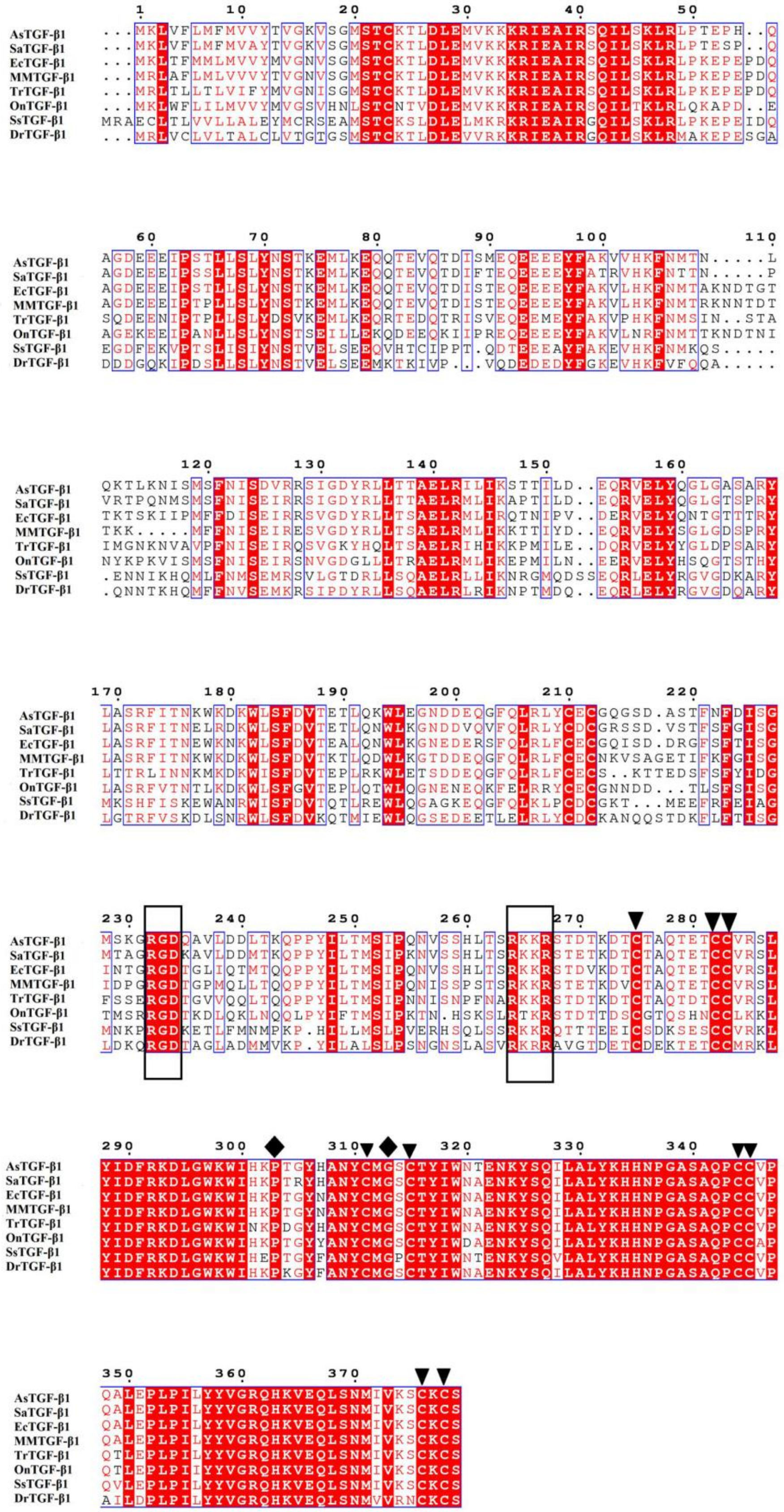

Supplementary Figure S1. Multiple alignment of the deduced amino acid (aa) sequences of TGF-β1. The multiple alignment was produced using ClustalW. The black box marked TGF-β1. RGD and RKRR sites of homologous amino acid sequences; "◆" marks proline and glycine, while "▼" represents cysteine residues.

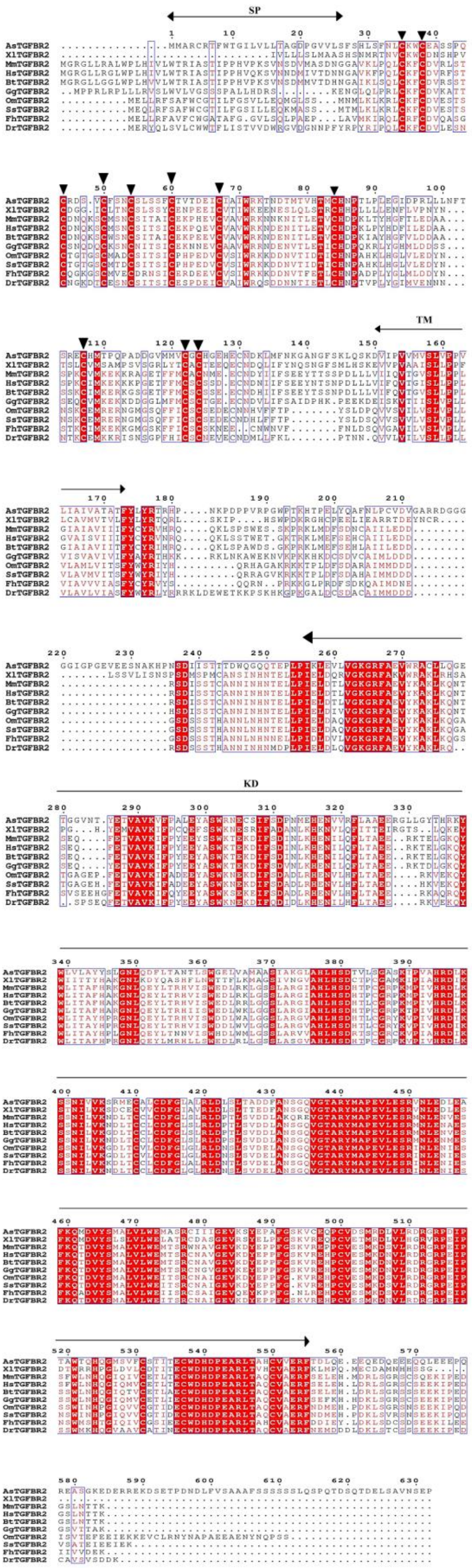

Supplementary Figure S2. Multiple alignment of the deduced amino acid (aa) sequences of TβR-II. The multiple alignment was produced using ClustalW.

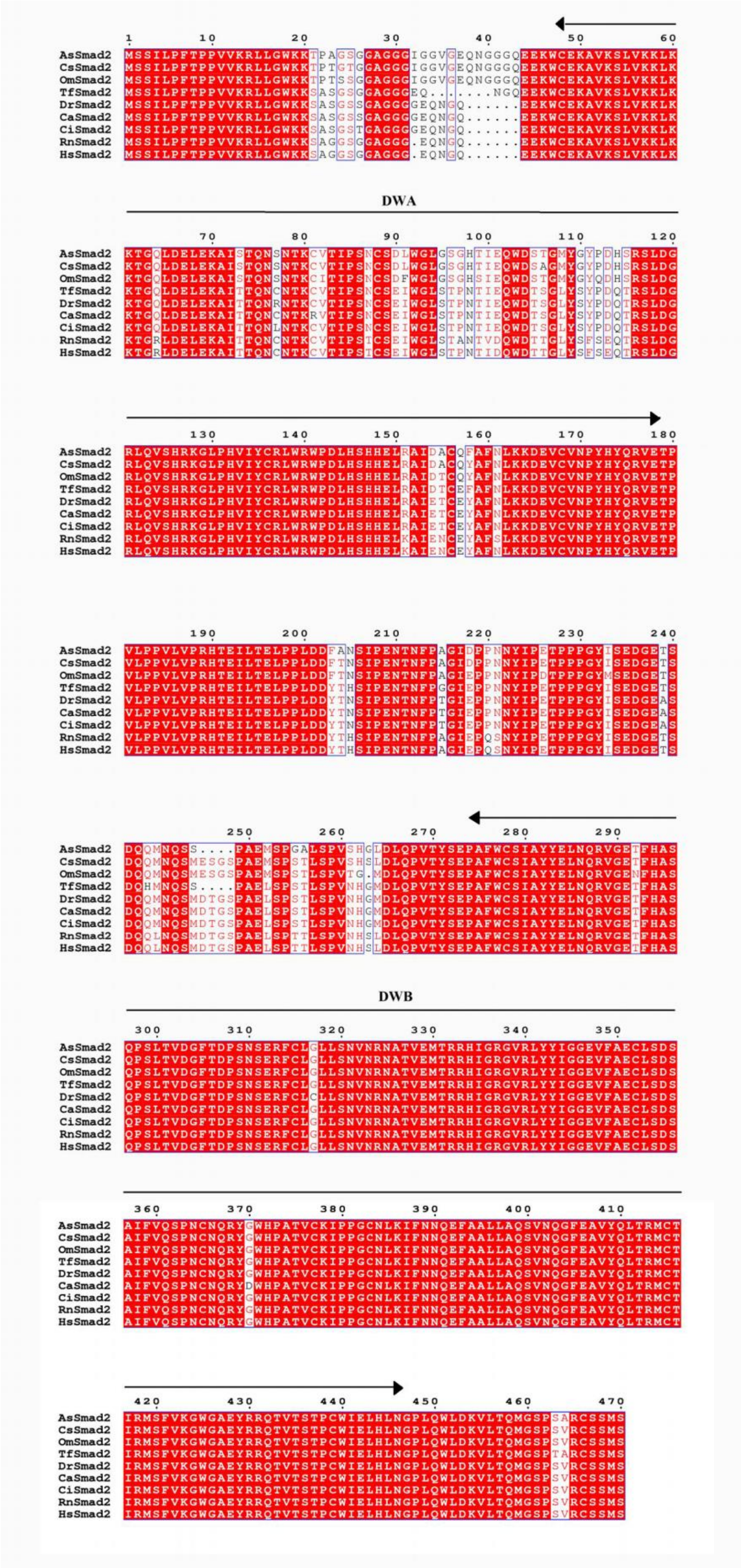

Supplementary Figure S3. Multiple alignment of the deduced amino acid (aa) sequences of Smad2. The multiple alignment was produced using ClustalW.

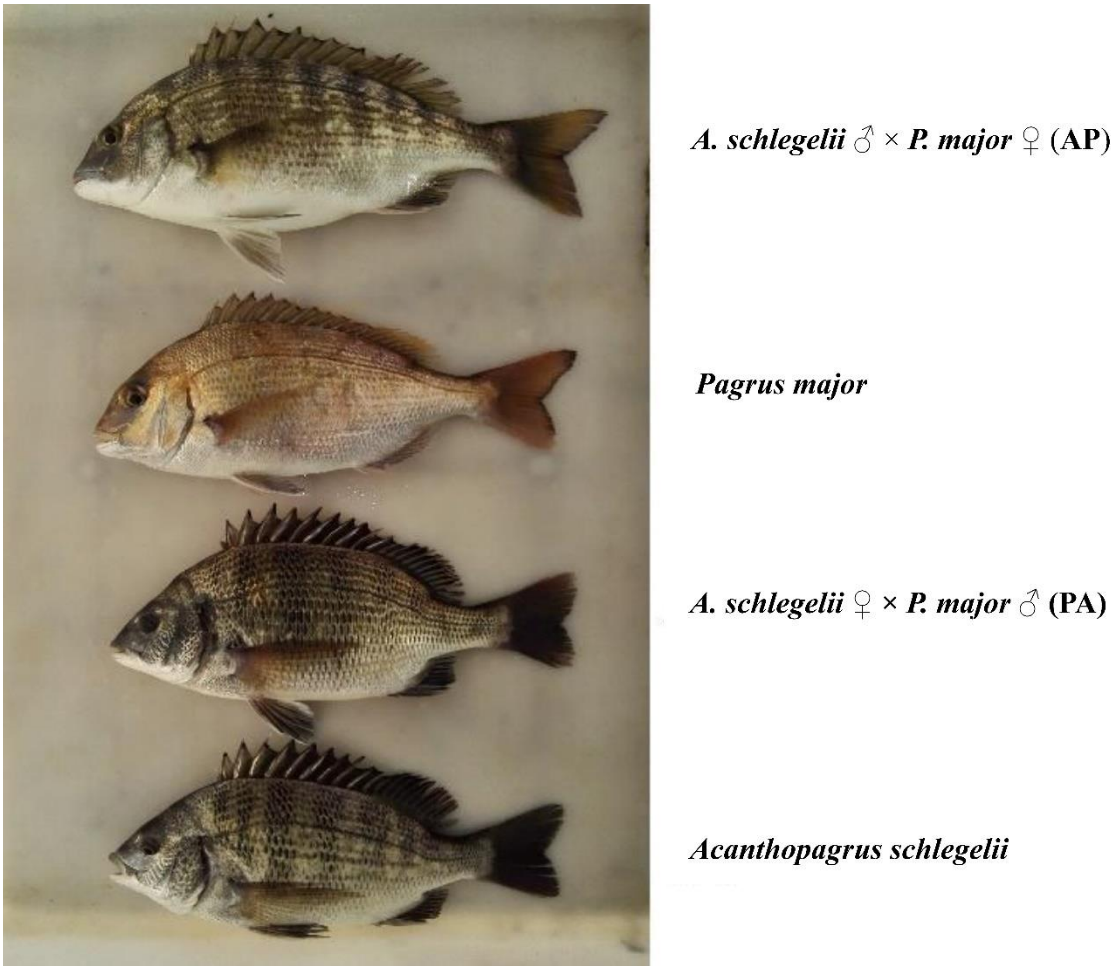

Supplementary Figure S4. Body shapes of *Acanthopagrus schlegelii*, *Pagrus major* and their hybrid offsprings [1]

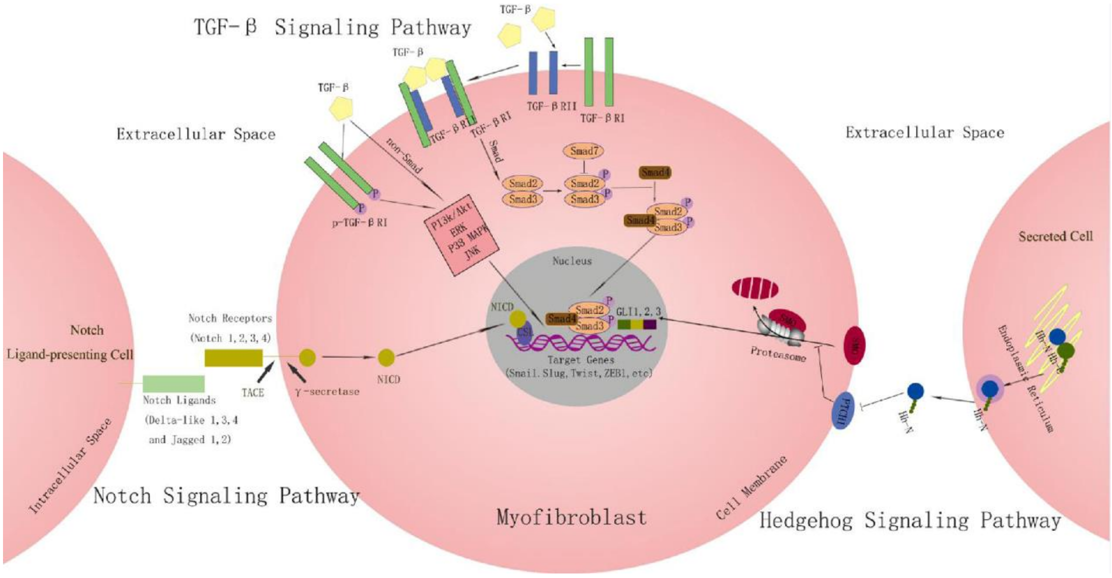

Supplementary Figure S5. Diagram of TGF-β1/Smads pathways [2]

1. Cao, G. Comparative research of genetic characterization in *Acanthopagrus schlegelii*, *Pagrus major* and their hybrid offsprings. Master's thesis, Shanghai Ocean University, 2021.
2. Chen, Y.; Fan, Y.; Guo, D. Y.; Xu, B.; Shi, X. Y.; Li, J. T.; Duan, L. F., Study on the relationship between hepatic fibrosis and epithelial-mesenchymal transition in intrahepatic cells. *Biomedicine & Pharmacotherapy* **2020,** *129*.
